# *Luteibacter jiangsuensis* blood stream infection: a first case report

**DOI:** 10.1186/s12879-023-08867-9

**Published:** 2023-12-07

**Authors:** Takanori Horiguchi, Makoto Sumiyoshi, Eri Nagatomo, Kazuki Sakamoto, Souichiro Ogawa, Naoki Ichinari, Akiteru Yamada, Yuki Rikitake, Chihiro Iwao, Takeshi Kawaguchi, Kunihiko Umekita, Ichiro Takajo, Shojiro Yamamoto, Taiga Miyazaki

**Affiliations:** 1 Division of Respirology, Rheumatology, Infectious Diseases, and Neurology, Department of Internal Medicine, Faculty of Medicine, University of Miyazaki, Miyazaki, Japan; 2 Division of Gastroenterology and Hematology, Department of Internal Medicine, Faculty of Medicine, University of Miyazaki, Miyazaki, Japan

**Keywords:** *Luteibacter Jiangsuensis*, Blood stream Infection, Bacteremia, DNA sequence

## Abstract

**Background:**

*Luteibacter jiangsuensis* is a gram-negative aerobic bacillus that was first isolated from soil samples at a pesticide factory in China and reported in 2011. Here, we describe the first case of *L. jiangsuensis* infection in human.

**Case presentation:**

A 59-year-old Japanese woman undergoing treatment for Crohn’s disease was admitted to our hospital with fever. Clinical examination indicated catheter-related bloodstream infection. The catheter was removed and meropenem was initiated. Morphologically identical glucose non-fermentative gram-negative bacilli were detected from two sets of aerobic blood culture and catheter-tip cultures. MALDI-TOF mass spectrometry failed to identify the bacterium, which was later identified as *L. jiangsuensis* by 16 S rRNA gene sequencing. Antimicrobial susceptibility test revealed that the isolate was resistant to carbapenem, therefore meropenem was switched to intravenous levofloxacin (500 mg/day). After 14 days of treatment with levofloxacin, the patient was discharged.

**Conclusions:**

This is the first case of *L. jiangsuensis* infection in human. The strain was identified by 16 S rRNA gene sequence analysis.

## Background

*Luteibacter jiangsuensis* is a gram-negative aerobic bacillus in the genus *Luteibacter* of the family *Rhodanobacteraceae*. *L. jiangsuensis* (strain JW-64-1^T^) was first isolated in 2011 from soil samples taken from a pesticide (methamidophos)-manufacturing factory in China [1], but there have been no reports of human *L. jiangsuensis* infections, and its pathogenicity to humans is unknown. We report the case of a catheter-related bloodstream infection (CRBSI) caused by *L. jiangsuensis* in a patient with Crohn’s disease.

## Case presentation

A 59-year-old Japanese woman who had been diagnosed with Crohn’s disease at age 24 was undergoing treatment with 6-mercaptopurine, mesalazine, and infliximab (300 mg/month). A subcutaneous central venous (CV) access port was placed in the patient’s anterior chest wall at age 53, and total parenteral nutrition had been continuously administered at home. The patient was a housewife with no notable history of occupation, hobbies, or international travel. Five days before her admission, the patient developed a > 40 ℃ fever and was admitted to our hospital for further evaluation.

**Fig. 1:**
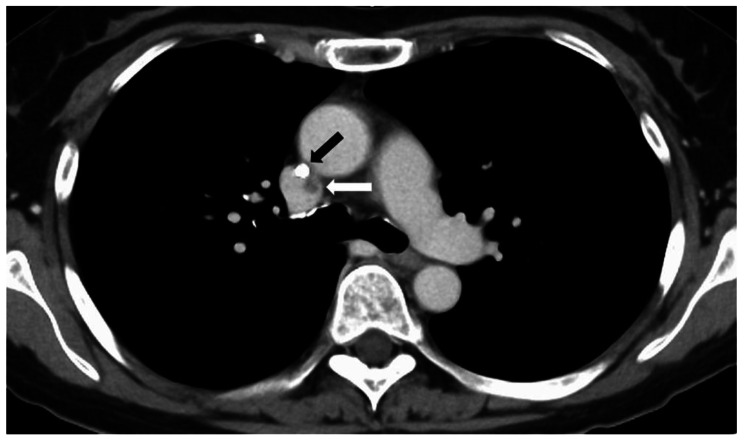
Contrast-enhanced computed tomography image on admission. Black arrow: central venous catheter placed in the superior vena cava. White arrow: a 10-mm-diameter hypodense lesion adjacent to the catheter

**Fig. 2:**
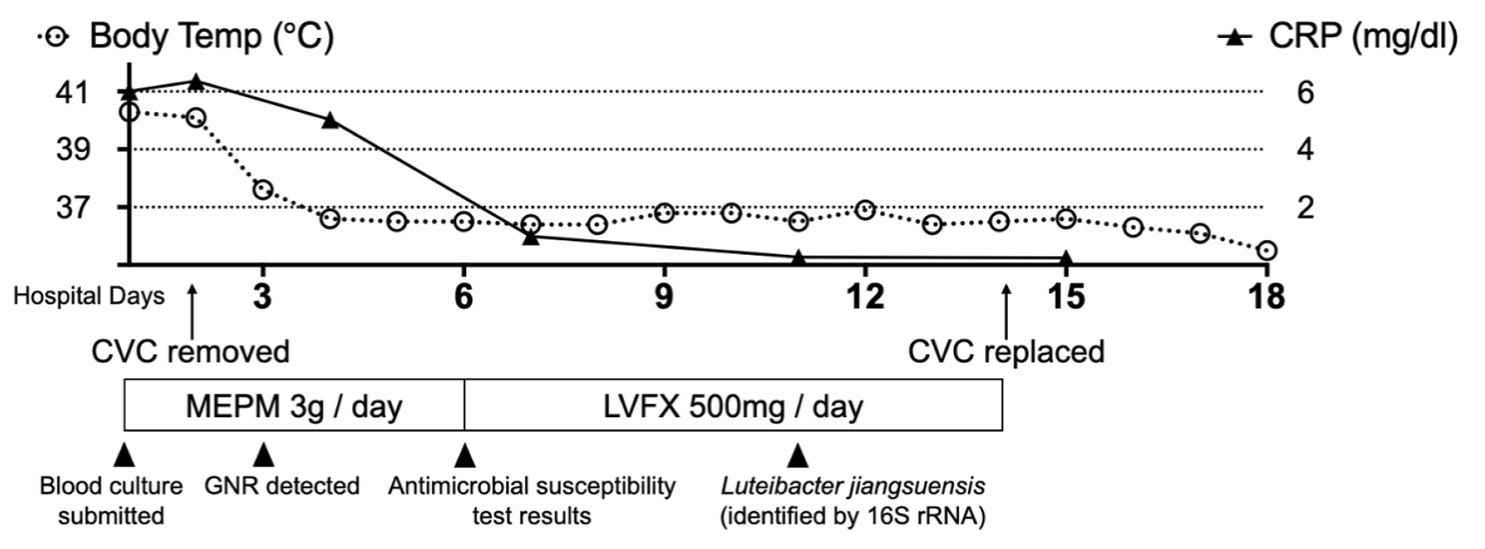
Clinical course. CRP: c-reactive protein, CVC: central venous catheter, MEPM: meropenem, LVFX: levofloxacin, GNR: gram negative rod

A physical examination revealed swelling and redness around the CV port insertion site. A contrast-enhanced computed tomography examination showed a 10-mm hypodense lesion at the tip of the CV catheter in the superior vena cava (Fig. 1). The results of blood tests were as follows: white blood cell count, 4.0 × 10^9^ /L; neutrophil percentage, 48.0%; absolute neutrophil count, 1.9 × 10^9^ /L; C-reactive protein, 60.1 mg/L; procalcitonin, 0.43 ng/mL; albumin, 3.6 g/dL; blood urea nitrogen, 9.9 mg/dL; and creatinine, 0.90 mg/dL. Two sets of aerobic and anaerobic blood cultures were submitted, and treatment was initiated with meropenem (1 g, thrice daily). On day 2 of hospitalization, the CV port/catheter was removed, and the catheter tip was submitted for two sets of culture testing. The patient’s fever drastically improved after initial treatment. On day 3, gram-negative rod bacteria were detected from both sets of aerobic blood culture at 31 and 36 h (anaerobic culture was negative). The same bacterium was also detected from the catheter-tip culture, and its morphology was suggestive of glucose non-fermentative bacilli. On day 6, drug susceptibility results (Table 1) revealed carbapenem resistance, therefore meropenem was switched to levofloxacin 500 mg/day by intravenous infusion. After 14 days of treatment with levofloxacin, CV port/catheter was placed, and the patient was discharged on day 18 of hospitalization (Fig. 2).

**Table 1:** Antimicrobial susceptibility of the *Luteibacter jiangsuensis* isolate

	MIC (mg/L)	SIR		MIC (mg/L)	SIR
ampicillin	> 16	–	cefditoren pivoxil	≦ 1	–
piperacillin	> 64	R	cefcapene pivoxil	≦ 1	–
ampicillin/sulbactam	16	–	cefmetazole	> 32	–
amoxicillin/clavulanic acid	≦ 8	–	flomoxef	> 32	–
piperacillin/tazobactam	≦ 16	S	latamoxef	≦ 8	–
cefoperazone/sulbactam	≦ 16	–	imipenem/cilastatin	8	I
cefazolin	> 16	–	meropenem	> 8	R
cefotiam	> 16	–	aztreonam	8	S
cefotaxime	32	I	gentamicin	≦ 4	S
ceftriaxone	8	S	tobramycin	≦ 4	S
ceftazidime	4	S	amikacin	≦ 16	S
cefozopran	≦ 8	S	minocycline	≦ 4	S
cefepime	≦ 2	S	levofloxacin	≦ 0.12	S
cefaclor	> 16	–	ciprofloxacin	≦ 0.25	S
cefpodoxime proxetil	≦ 2	–	sulfamethoxazole-trimethoprim	≦ 2	S

MICs were determined using the broth microdilution method. The SIR was determined using CLSI breakpoints (glucose non-fermenting bacteria)

**Fig. 3:**
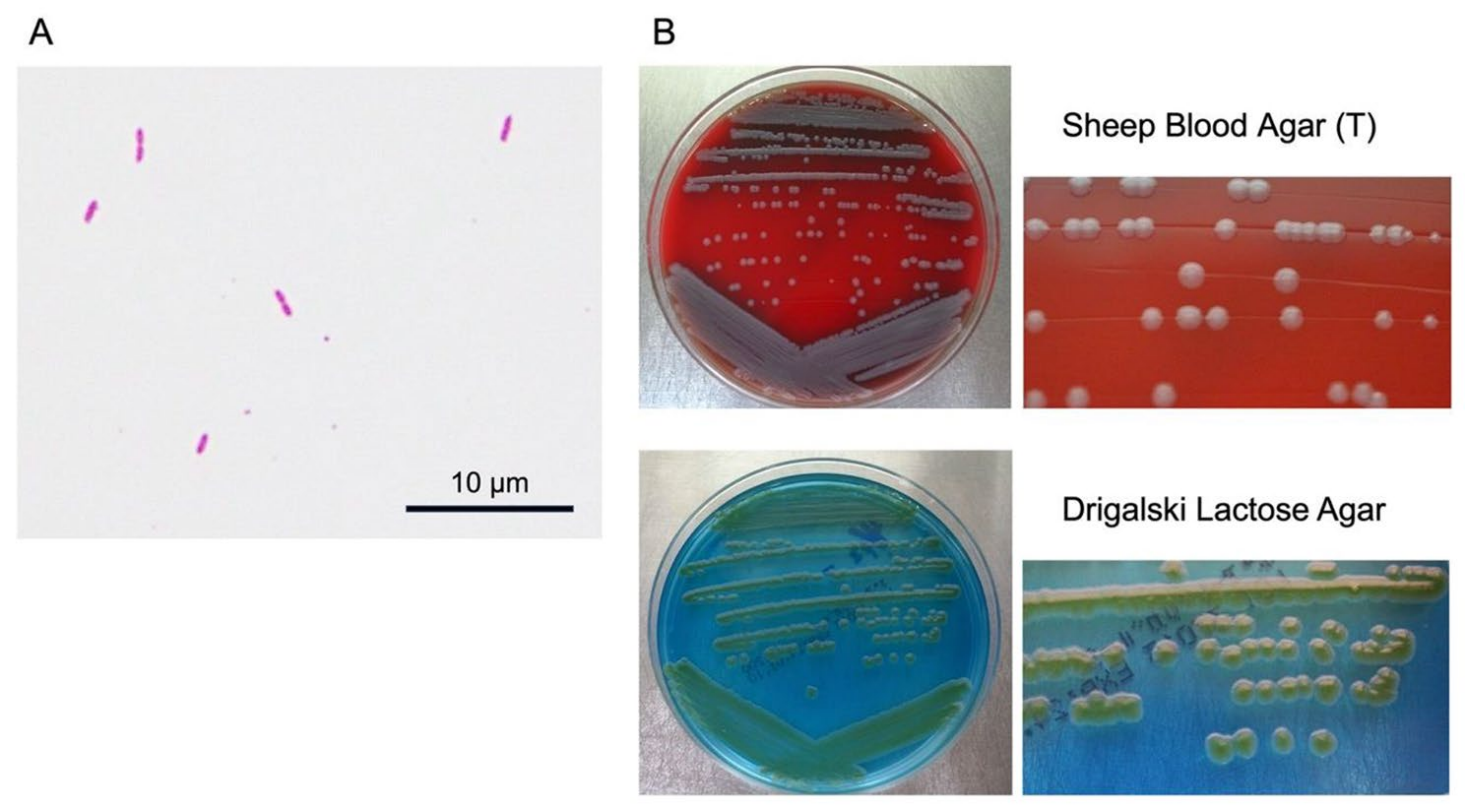
Morphological features of the *Luteibacter jiangsuensis* isolate. **A**: Gram Stain. Gram-negative, aerobic, spore-free, rod-shaped bacteria. Scale bar, 10 μm. **B**: Colonies of the *L. jiangsuensis* isolate on Sheep Blood Agar (T) and Drigalski Lactose Agar

Regarding the identification of bacterial species, the top 3 matches and scores given by Matrix-assisted laser desorption/ionization-time of flight mass spectrometry (MALDI-TOF MS) were *Burkholderia caribensis* (score 1.209), *Raoultella ornithinolytica* (score 1.198), and *Arthrobacter globiformis* (score 1.187). Because the score was below 1.7, species of the isolate could not be identified by MALDI-TOF MS. Later, the isolate was identified as *L. jiangsuensis* by 16 S rRNA gene sequencing. The *L. jiangsuensis* isolate was Gram-negative, aerobic, spore-free, rod-shaped bacteria (Fig. 3A) and produced circular, smooth, grey-colored colonies (2–3 mm) on Sheep Blood Agar (T) and green-colored colonies (3–4 mm) on Drigalski Lactose Agar after 24 h incubation (Fig. 3B). Biochemical characteristics of the strain isolated from the present patient were similar to those of strain JW-64-1^T^ except for catalase activity, acid production from glucose and lactose, and nitrate reduction (Table 2). Although strain JW-64-1^T^ can reduce nitrate to nitrite, our strain could not reduce nitrate consistent with other *Luteibacter* spp. [1]. Antimicrobial susceptibility test of the isolate revealed high minimum inhibitory concentration (MIC) values for first to third generation cephalosporins and carbapenems (Table 1).

**Table 2:** Biochemical characteristics of the *Luteibacter jiangsuensis* isolate

	This study	Wang et al. [1]
Oxidase^1^	+	+
Catalase^2^	–	+
Voges Proskauer^3^	–	N/A
Motility^4^	–	–
_D_-Glucose	+	–
_D_-Fructose	+	N/A
Maltose	+	+
_D_-Galactose	+	N/A
_D_-Xylose	+	+
_D_-Mannitol	–	N/A
Sucrose	–	N/A
Lactose	–	+
Esuclin	+	N/A
Urease	–	–
Citrate	–	N/A
β-Galactosidase	+	+
Lysine Decarboxylase	+	+
Arginine Dihydrolase	+	+
Ornithine Decarboxylase	+	+
Indole	–	–
Nitrate reduction	–	+
Gelatinase	+	+

All items were evaluated using ID test NF-18 (Shimadzu Diagnostics, Tokyo, Japan) in this study except the followings

^1^Evaluated using Cytochrome Oxidase Test Strip (Shimadzu Diagnostics)

^2^ Evaluated using 3% hydrogen peroxide (Fujifilm Wako Chemicals, Osaka, Japan)

^3^Evaluated using VP test (Eiken Chemical, Tokyo, Japan)

^4^Evaluated using sulfide indole motility medium (Eiken Chemical)

## Discussion and conclusions

We report the first case of CRBSI caused by *L. jiangsuensis*. The genus *Luteibacter* was first reported and established by Johansen et al. in 2005 [2]. Although the genus was originally thought to belong to the family *Xanthomonadaceae* in the class *Gammaproteobacteria*, genomic analysis revealed that it is in the family *Rhodanobacteraceae* [3]. To our knowledge, only two clinical cases of the genus *Luteibacter* infection in humans have been reported: CRBSI caused by *L. anthropi* [4] and a *Luteibacter* sp., which shared 97.4% identity with *L. rhizovicina* [5]. The present case is the first report of *L. jiangsuensis* infection in humans.

*Luteibacter* spp. is found primarily in environmental soil. *L. jiangsuensis* has the capacity to grow at 4–42 ℃ (optimum temperature 37 ℃) and pH 4.5–8.0 (optimum pH 7.0). *L. jiangsuensis* can hydrolyze and utilize substrates of organic compounds, including carbohydrates. However, the pathogenicity of *L. jiangsuensis* in mammals is unknown due to the absence of clinical and experimental data. The patient in this report had no exposure to soil or any other suspected sources of infection. Like our present patient, the individual with bacteremia caused by a *Luteibacter* sp. also had CV catheter and was immunocompromised due to hematological disorder and chemotherapy [5]. We thus speculate that malnutrition from Crohn’s disease, the use of immunosuppressive drugs, and CV catheter placement may have been the risk factors for our patient’s *Luteibacter* bacteremia.

In vitro susceptibility testing showed that the isolate was carbapenem resistant, yet treatment with meropenem appeared to be effective (Fig. 2). The removal of CV port/catheter was considered a major contributor to this favorable clinical course. The clinical breakpoints and the epidemiological cutoff values for *Luteibacter* spp. have not been established by the Clinical and Laboratory Standards Institute (CLSI) or the European Committee on Anti-microbial Susceptibility Testing. Therefore, we used the SIR breakpoints determined for “glucose non-fermenting bacteria” in CLSI. In the future, further studies including whole genome sequencing should be conducted to elucidate the mechanism of drug resistance of this isolate.

## Materials and methods

### Gram staining, culture conditions, and antimicrobial susceptibility testing

Gram staining was performed using the neo-B&M Wako (Fujifilm Wako Chemicals, Osaka, Japan). The *L. jiangsuensis* isolate was grown at 37 ℃ for 24 h on Sheep Blood Agar (T) (Nippon Becton Dickinson, Tokyo, Japan) and Drigalski Lactose Agar (Eiken Chemical, Tokyo, Japan).

For antimicrobial susceptibility testing, bacterial strains were incubated at 35 ℃ for 18 h and minimal inhibitory concentrations (MICs) were determined using MicroScan WalkAway-96 plus with Neg MIC 3 J panel (Beckman Coulter, CA, USA) according to the manufacturer’s instruction. The SIR (susceptible/intermediate/resistant) was determined according to the criteria of CLSI M100-30th edition for glucose non-fermenting bacteria [6].

### Determination of biochemical characteristics of the *Luteibacter jiangsuensis* isolate

Oxidase activity was evaluated using Cytochrome Oxidase Test Strip (Shimadzu Diagnostics, Tokyo, Japan). Catalase activity was evaluated using 3% hydrogen peroxide (Fujifilm Wako Chemicals, Osaka, Japan) as described previously [7]. Voges Proskauer and motility were examined using VP test (Eiken Chemical, Tokyo, Japan) and sulfide indole motility medium (Eiken Chemical), respectively. Other items were evaluated using ID test NF-18 (Shimadzu Diagnostics). These tests were performed according to the manufacturer’s instructions.

### Matrix-assisted laser desorption/ionization- time of flight mass spectrometry (MALDI-TOF MS)

Bacteria were cultured on Sheep Blood Agar (T) at 35 ℃ for 18 h. The colony was then suspended in purified water and adjusted to a turbidity of McFarland 0.5 (estimated number of bacteria: 1.5 × 10^8^ CFU/ml). Fifty microliters of the bacterial suspension were added to an equal volume of 50 mM NaOH and heated at 98 ℃ for 5 min. The resulting solution was neutralized with 100 µl of 100 mM Tris-HCl (pH 7.0) and used as a template for polymerase chain reaction (PCR). In accordance with the manufacturer’s instructions, the isolates were analysed by mass spectrometry using MALDI Biotyper™ (Bruker Daltonics, Bremen, Germany). Colonies were applied to Micro Scout Plate 48 target polished steel Barcode (Bruker Daltonics). One drop of matrix (α-cyano-4-hydroxycinnamic acid) including 2-cyan-3 (4-hydroxyphenyl) acrylic acid (Bruker Daltonics), was added. MALDI-TOF MS analysis was performed using Bruker Biotyper 3.1 software, in accordance with the instruction manual. The bacterium could not be identified by MALDI-TOF MS.

### 16 S rRNA gene sequencing

For 16 S rRNA gene amplification, DNA polymerase (Takara-Bio, Otsu, Japan) and the following primers were used: forward primer (1 F: 5′-AGAGTTTGATCMTGGCTCAG-3′ positions 1–20) [8] and reverse primer (1517R: 5′-TACGGTTACCTTGTTACGAC-3′ positions 1517–1498) [9]. PCR was performed based on the methods described previously [8, 9] using 2 µl of bacterial solution samples under the following conditions: one cycle at 98 ℃ for 20 s and then 25 cycles of denaturing at 98 ℃ for 10 s, annealing at 55 ℃ for 20 s, and extension at 72 ℃ for 30 s. PCR products were purified by ethanol precipitation. The DNA sequence was analysed by Applied Biosystem 3500 Genetic Analyzer (Thermo Fisher Scientific, Waltham, USA) using Big Dye™ Terminator V.3.1 Cycle Sequencing Kit (Thermo Fisher Scientific). For species confirmation, the amplicon sequences were assembled and then compared to reference sequences and other entries in the EZBioCloud (http://www.ezbiocloud.net/identify). EZBioCloud revealed 99.8% identity (1,405/1,416 bp) with *L. jiangsuensis* (family Rhodanobacteraceae; GenBank accession number ASM1174255v1) (Table 3).

**Table 3:** 16 S rRNA gene sequencing of the *Luteibacter jiangsuensis* isolate

Hit taxon Name	Hit strain name*	Accession no.*	Similarity	Variation ratio
*Luteibacter* *jiangsuensis*	JW-64-1 (T)	FJ848571	99.80	3/1467

*Reference strain from EZBioCloud (http://www.ezbiocloud.net/identify)

## Data Availability

The datasets used and/or analysed during the current study are available from the corresponding author on reasonable request.

## Abbreviations

CLSI: Clinical and Laboratory Standards Institute
CRBSI: Catheter-related bloodstream infection
CV: Central venous
MALDI-TOF MS: Matrix-assisted laser desorption/ionization- time of flight mass spectrometry
MIC: Minimum inhibitory concentration
PCR: Polymerase chain reaction
